# Rationale and design of a double-blind, placebo-controlled, randomized trial to evaluate the safety and efficacy of nimodipine in preventing cognitive impairment in ischemic cerebrovascular events (NICE)

**DOI:** 10.1186/1471-2377-12-88

**Published:** 2012-09-05

**Authors:** Penglian Wang, Yongjun Wang, Tao Feng, Xingquan Zhao, Yong Zhou, Yilong Wang, Weixiong Shi, Yi Ju

**Affiliations:** 1 Department of Neurology, Beijing Tiantan Hospital, Capital Medical University, Beijing, China

## Abstract

**Background:**

Stroke is the second most common cause of mortality and the leading cause of neurological disability, cognitive impairment and dementia worldwide. Nimodipine is a dihydropyridinic calcium antagonist with a role in neuroprotection, making it a promising therapy for vascular cognitive impairment and dementia.

**Methods/design:**

The NICE study is a multicenter, randomized, double-blind, placebo-controlled study being carried out in 23 centers in China. The study population includes patients aged 30–80 who have suffered an ischemic stroke (≤7 days). Participants are randomly allocated to nimodipine (90 mg/d) or placebo (90 mg/d). The primary efficacy is to evaluate the level of mild cognitive impairment following treatment of an ischemic stroke with nimodipine or placebo for 6 months. Safety is being assessed by observing side effects of nimodipine. Assuming a relative risk reduction of 22%, at least 656 patients are required in this study to obtain statistical power of 90%. The first patient was recruited in November 2010.

**Discussion:**

Previous studies suggested that nimodipine could improve cognitive function in vascular dementia and Alzheimer’s disease dementia. It is unclear that at which time-point intervention with nimodipine should occur. Therefore, the NICE study is designed to evaluate the benefits and safety of nimodipine, which was adminstered within seven days, in preventing/treating mild cognitive impairment following ischemic stroke.

## Background

It is well established that stroke contributes to about 5.4 million deaths per year and is the leading cause of neurological disability worldwide [1,2]. Cerebrovascular disease is now one of the main diseases affecting the health of Chinese people [3,4]. Motor and sensory impairment, cognitive impairment and even post-stroke dementia can be induced by stroke. Cognitive impairment and dementia following stroke can severely lower the ability of perform daily activities and decrease quality of life (QOL) in patients with stroke, and increase the rate of disability and death.

Cognitive impairment was proposed as a prognostic index of stroke by the Hachinski group in 2007 [5]. Subclinical stroke and leukodystrophy may cause some cognitive changes in older patients, such as disturbances of thinking and reasoning, disturbances of memory and recall of recent events, and disorders of social behavior and depression [6,7]. A population-based study in Japan observed that 42.5% of early-onset dementia cases were related to vascular diseases [8]. Previous studies comparing patients with and without a stroke found that stroke was strongly associated with incident dementia [9-13]. Thus, prevention is crucial for both reduction of frequency of post-stroke cognitive decline and dementia and decreasing economic burden following stroke.

The worldwide therapeutic model for stroke treatment is transforming from a focus on improvement of somatic function to improving the whole QOL, including preventing and treating post-stroke dementia or vascular cognitive impairment (VCI) [14]. Post-stroke cognitive impairment consists of vascular dementia, mixed dementia and non-dementia cognitive impairment [15,16]. Vascular dementia can be been treated with cholinesterase inhibitors (Donepezil and Rivastigmine), N-methyl d-aspartate (NMDA) receptor antagonists (Memantine) and some nootropic drugs. However, previous studies showed that cholinesterase inhibitors had controversial roles in treating vascular diseases [17-19]. Memantine had only a small effect on improving cognitive impairment [20]. In addition, previous studies did not supply any supportive evidence for the effect of Piracetam on vascular cognitive impairment [21]. So far, clinical trials on the pharmacotherapy of vascular dementia for the above-mentioned drugs have yielded unsatisfactory results. In contrast, an evidence-based meta-analysis demonstrated that nimodipine could improve cognitive function in vascular dementia, Alzheimer's disease (AD) dementia and mixed dementia with good tolerance [22]. Also, vascular dementia can be partly induced by altering intracellular calcium metabolism, although the mechanisms underlying the impact of cerebrovascular diseases on vascular dementia are not fully understood [23-25]. Therefore, the evidence is needed to choose a valid option for preventing and treating cognitive impairment after a stroke.

## Rational and discussion

Nimodipine is a dihydropyridinic calcium antagonist that quickly and easily crosses the BBB and reaches a high concentration in cerebrospinal fluid (CSF). It improves cerebral perfusion after acute ischemia [26] and reduces neurological deficits [27-29]. It has specific affinity for receptor operated calcium channels in cerebral vessels and for specific membrane-located receptor sites that may be associated with the pharmacological action of nimodipine in the brain [30,31]. Results from in vivo research showed that nimodipine also has a high affinity for specific binding sites in the cortex, the dentate gyrus and the hippocampus, which are known to be the core regions involved in cognitive function [32]. Based on this evidence, many studies have tried to elucidate the role of nimodipine in treating cognitive impairment. A published Meta-analysis from 14 randomized, double-blind and control clinical trials (3166 cases) suggested that nimodipine (90 mg/d, 12–26 weeks) could improve the cognitive deficits caused by unclassified disease, Alzheimer’s disease, cerebrovascular disease, or mixed Alzheimer’s and cerebrovascular disease [22]. A wide variety of scales, such as the mini-mental state examination (MMSE), short cognitive performance test (SKT), clinical global impression (CGI), Sandoz clinical assessment geriatric scale (SCAG) and Alzheimer’s disease assessment score-cognitive component (ADAS-cog), were adopted to evaluate cognitive impairment in this Meta-analysis. The results suggested that nimodipine could significantly delay the decrease in SCAG score [WMD (weighted mean difference) -11.75, 95% CI −15.64–-7.85, *P* < 0.00001] and CGI (WMD −1.31, 95% CI −1.73–-0.89, *P* <0.00001) at 12 weeks following stroke [22].

The first randomized, double-blind, controlled trial focusing on subcortical vascular dementia and multiple infarction dementia presented the results in 2005 [33]. This study noted a 22.4% reduction in deterioration (3 or more point-drop versus baseline) on the MMSE within a 52-week period in nimodipine-treated patients when compared with the placebo group, who had similar demographic characteristics. There was also a significant delay in the deterioration of SCAG scores and significant improvement in SCAG scores.

The pivotal principal of cognitive impairment prevention following stroke is to intervene early rather than cure late. An important study in Hong Kong (China) was carried out to examine the effectiveness of nimodipine prevention/therapy on cognitive impairment in acute cerebral infarction patients, with the aim of demonstrating the effectiveness of early intervention after stroke [34]. Although this study was a single-blind randomized controlled trial and enrolled 100 patients, promising results were found in acute cerebral infarction patients. In the intervention patients, Fuld object-memory evaluation (FOME) mean scores were significantly improved and the death rate did not increase within a 12-week period.

An important consideration is at which time-point intervention with nimodipine should occur. This varies between studies, as most are administered at a chronic stage (i.e. >6 months after the stroke) and 2 weeks after the stroke [22,35]. However, a series of chain reactions induce a large amount of neuronal damage at acute and subacute stages of stroke [36]. Theoretically, nimodipine could have clinical benefits due to its ability to modulate blood vessels and protect neurons, especially its probable ability to regulate the capillary with relatively intact structure and functions in the severely injured brain [27,30,37,38]. It is thus conceivable that it is clinically beneficial to begin nimodipine therapy at early stage after the stroke.

The aim of this study is therefore to evaluate the clinical efficacy and safety of nimodipine, administered within seven days after an acute ischemic stroke, in the prevention of mild cognitive impairment.

## Methods/design

### Study design

**Figure 1:**
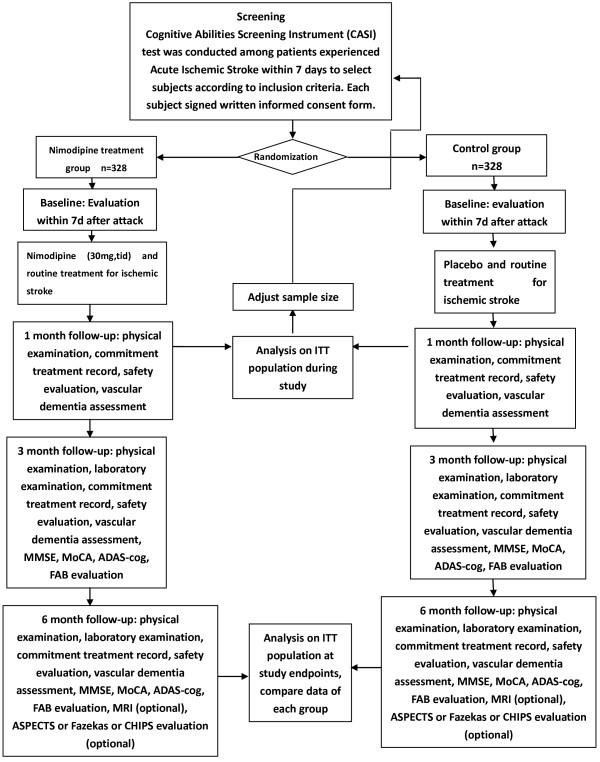
**The NICE study flowchart.** The intention-to-treat (ITT).

The NICE study is a phase IV clinical designed as a multicenter, randomized, double-blind, placebo-controlled, parallel-group study, which is being carried out in 23 centers in China (Additional file 1: Appendix). The study design is presented in Figure 1. The inclusion criteria and major exclusion criteria are shown as the following.

The inclusion criteria are:

Male or female, aged 30-80;

Acute ischemic stroke diagnosed according to ICD-10 and CT/MRI criteria;

≤7d after the stroke;

MMSE score > Dementia threshold corrected based on educational year (>17 scores, >20 scores and >24 scores for illiterate, primary school, and above middle school respectively);

MoCA <26 scores for >12 educational years (or <25 scores for ≤12 educational years);

Hachinski ≥7 scores;

Expected good compliance with the therapy and follow-up. Already obtained written informed consent form.

Major exclusion criteria are:

Pregnant or lactating women;

History of mental diseases (eg. Schizophrenia and serious anxiety depression);

Patients with AD, Parkinson's disease, frontotemporal dementia or Huntington's disease (including the existing dementia diagnosed before the stroke or the past definite cognitive impairment);

Dementia of other causes (eg. injury of central nervous system, tumor, infection, metabolic disease, hydrocephalus at normal pressure, deficiency of folic acid/vitamin B12 and hypothyroidism);

Patients with other influencing factors for cognition evaluation (eg. aphasia, serious auditory/visual impairment and hemiplegia at dominant side);

Patients with contraindications for dihydropyridines;

Patients with serious arrhythmia, heart rate > 120 bpm (or < 50 bpm); ever myocardial infarction within 6 months; BP < 90/60 mmHg; serious heart failure (unable to engage in any physical activity, and dyspnea and cardiac edema or angina pectoris at rest);

Patients having serious kidney incompetence: Creatinine >1.5 times of upper limit of normal value (ULN);

Patients with serious liver incompetence: AST/ALT level >3 times of ULN;

History of known allergy;

History of blood coagulation dysfunction, hemorrhagic disease, thrombocytopenia, leukocytopenia or neutropenia, serious anemia, Hb < 100 g/L;

History of serious gastrointestinal disease;

Patients with any known malignant tumor;

Patients with epilepsy and/or ever having antiepileptic drugs.

The diagnosis of acute ischemic stroke is determined by clinical examination and magnetic resonance imaging (MRI). The threshold of MMSE scores is corrected based on years of education (>17 scores, >20 scores and >24 scores for illiterate, primary school, and above middle school respectively) [39]. Montreal cognitive assessment (MoCA) scores are <26 for >12 educational years, and <25 for ≤12 educational years [40]. Hachinski scores are ≥ 7 [41].

### Ethics and informed consent

The study is being performed in accordance with the ethical principles proclaimed in the Declaration of Helsinki in 1964, and revised in Tokyo in 2004. The study protocol has been reviewed by independent ethics committees in China, and by coordinators, investigators, and sponsors in accordance with local regulations. According to local requirements, thorough study information is provided to patients and written informed consent is obtained from each patient or surrogate family member before inclusion in the study. The NICE study is registered with clinicaltrials.gov (registration number: NCT01220622).

### Treatment and follow-up

Inclusion and exclusion criteria are verified and it is decided for each patient whether the study drug can be administered within 7 days after the onset of ischemic stroke. Patients are given nimodipine, 30 mg, tid, three tablets daily, or placebo, 30 mg, tid, three tablets daily. Intervention allocation is based on balanced, randomized, permuted blocks which were produced by a third party statistician who was blinded to the whole protocol. The study interventions are identical in appearance, and investigators and patients are blinded to intervention allocation. Aside from basic treatment for acute ischemic stroke, cholinesterase inhibitors, NMDA receptor antagonists, or drugs from the racetam family cannot be used in this study. The following information is collected at the baseline assessment:

(1) Demographic data;

(2) Blood pressure, temperature, and pulse rate;

(3) Time from stroke onset;

(4) Medical history;

(5) Previous and ongoing medications;

(6) Assessing vascular dementia according to National Institute of Neurological Disorders and Stroke, -and the Association Internationale pour la Recherche et l’Enseignement en Neurosciences (NINDS-AIREN) criteria;

(7) NIH stroke scale (NIHSS), ADAS-cog, Frontal assessment battery (FAB), Alberta stroke program early CT score (ASPECTS), Fazekas, The cholinergic pathways hyperintensities scale (CHIPS).

Follow-up visits are carried out at 1, 3, and 6 months (Figure 1). A physical examination is performed, and concomitant treatments are recorded at every visit. Compliance is determined by counting tablets at every visit. Cognitive function is assessed using MMSE, MoCA, ADAS-cog, and FAB, at 3 and 6 months.

Safety is evaluated at every visit, including adverse events (AE) and serious adverse events (SAE), regardless of whether they are related to the administration of the study drug or course of therapy. All AE or SAE are reported and followed up completely. Laboratory tests are collected at 3 and 6 months. Patients who break off study intervention and informally withdraw consent will be followed up for the duration of the study.

### Primary outcome

The primary efficacy is defined as the combined results of the following efficacy criteria:

(1) MMSE (0–30)

(2) ADAS-cog (0–70)

MMSE is widely used for assessing cognitive function [39,42], and ADAS-cog also includes items used in evaluating cognitive function [43,44]. These two criteria have high reliability, and have been used in other stroke trials [22,33-35,44]; therefore the validation of the study results is possible.

The primary end-point for evaluating the efficacy of nimodipine for prevention/treatment of cognitive impairment is 6 months after acute ischemic stroke events.

### Secondary outcome

The secondary study end-points include the following analysis:

(1) the efficacy criteria of MoCA (0–30) and FAB (0–18) at 3 and 6 months;

(2) the efficacy criteria of MMSE and ADAS-cog at 3 months;

(3) the individual items of ADAS-cog, MoCA and FAB at 3 months;

(4) the individual items of ADAS-cog, MoCA and FAB at 6 months;

(5) the time course of MMSE, ADAS-cog, MoCA and FAB;

(6) overall mortality.

### Statistical analysis

In this multicenter trial, we plan to enroll approximately 656 patients, with approximately 328 patients per study group. Approximately 23 centers in China are participating, with each center enrolling about 30 patients. We assumed that there was no significant difference between nimodipine (90 mg/d) and placebo (90 mg/d) in the prevention/treatment of aggravated cognitive impairment of acute ischemic stroke in the 6 month follow-up. For a statistical power of at least 90% in this trial, we estimated that 656 patients are necessary to present the superiority of nimodipine versus placebo. This corresponds to a relative risk (RR) reduction of 22% (RR is 0.78 for the nimodipine group), using a two-sided test (α = 0.05, allowing 5% dropout). The efficacy analysis is based on an intention-to-treat method. Missing values are still treated as missing. Patients are examined at the last follow-up (at the occurrence of clinical event, the end of study, or the last follow-up before drop-out). A Kaplan-Meier curve is used to simulate the cumulative risk for cognitive impairment in the follow-up at day 180. The Cox proportional hazard model is used to calculate hazard proportion and 95% confidence interval. Log-rank test is used to evaluate the efficacy.

Most secondary outcome analyses will adopt the strategy of the primary outcome analyses. Continuous variables (for example MoCA and FAB) will undergo multi-variate linear regression analysis. Converted and/or weighted least square methods are regarded as abnormal and heterogeneous variance of remedial measures. Limit values will undergo a validity test. The conclusion will undergo sensitivity analysis.

### Study organization

The NICE trial is headed by a steering committee (Additional file 1: Appendix), which is assisted by an independent data safety and monitoring board (DSMB). Trial monitoring and data management is performed by Giant Med-Pharma Service Group. The Steering Committee will regularly review the status of the trial and available blinded data, and will perform appropriate actions according to the conduct of the study. A face-to-face Executive Committee meeting will be required to make major decisions. To ensure the study meets the highest standards of ethics and patient safety, the Safety and Monitoring Board will meet regularly and monitor the progress of the study. The Board is composed of Academic Members, including an independent statistician, who are not otherwise participating in the trial. Clinical end-point events (stroke, myocardial infarction, death, and overt bleedings) will be reviewed by independent experts (neurologists, cardiologists) in the Critical Events Committee. The sponsor of the NICE trial is the Minister of Science and Technology of the People's Republic of China. The sponsor has no influence on the study design, data collection, data analysis, and final drafting of this manuscript.

## Conclusion

The primary objective of this phase IV multicenter double-blind, placebo-controlled, randomized clinical trial is to prove the efficacy of nimodipine in the prevention/treatment of mild cognitive impairment following acute ischemic stroke. Approximate 23 centers in China are enrolled in the NICE trial, and the first patient was recruited into the study in November 2010. This trial analyses the safety of nimodipine and investigates several secondary outcomes. The results of the NICE study are expected in 2013.

## Abbreviations

AD: Alzheimer’s disease; ADAS-cog: Alzheimer’s disease assessment score-cognitive component; AE: Adverse event; ALT: Alanine transaminase; AST: Aspartate transaminase; ASPECTS: Alberta stroke program early CT score; BP: Blood pressure; CGI: Clinical global impression; CHIPS: The cholinergic pathways hyperintensities scale; CSF: Cerebrospinal fluid; DSMB: Data safety and monitoring board; FAB: Frontal assessment battery; FOME: Fuld object-memory evaluation; Hb: Hemoglobin; ICD-10: International classification of diseases, tenth revision; ITT: The intention-to-treat; MMSE: Mini-Mental state examination; MoCA: Montreal cognitive assessment; MRI: Magnetic resonance imaging; NICE: Nimodipine in Preventing Cognitive Impairment in Ischemic Cerebrovascular Events; NIHSS: NIH stroke scale; NINDS-AIREN: National Institute of Neurological Disorders and Stroke, -and the Association Internationale pour la Recherche et l’Enseignement en Neurosciences; NMDA: N-methyl d-aspartate; QOL: Quality of life; RR: Relative risk; SAE: Serious adverse event; SCAG: Sandoz clinical assessment geriatric scale; SKT: Short cognitive performance test; ULN: Upper limit of normal value; VCI: Vascular cognitive impairment; WMD: Weighted mean difference.

## Competing interests

The authors declare that they have no competing interests.

## Authors’ contributions

PW drafted the manuscript. YW1 and TF participated in the study design and helped to draft the manuscript. XZ participated in the study coordination and revised partially the important part of the manuscript. YZ and YW2 participated in statistics of the study data. WS and YJ participated in revising the design part of the manuscript. All authors read and approved the final manuscript.

## Pre-publication history

The pre-publication history for this paper can be accessed here:

http://www.biomedcentral.com/1471-2377/12/88/prepub

## Supplementary Material

Additional file 1 Appendix. Click here for file
