# Supplementary material for: Rationale and design of a double-blind, placebo-controlled, randomized trial to evaluate the safety and efficacy of nimodipine in preventing cognitive impairment in ischemic cerebrovascular events (NICE)
Source: BMC Neurol. 2012 Sep 5;12:88. doi: 10.1186/1471-2377-12-88 (PMC3488311; doi:10.1186/1471-2377-12-88)
Supplement: Additional file 1 — Appendix. [file 1471-2377-12-88-S1.doc]

**Appendix**

Principle Investigators: Yongjun Wang, Beijing Tian Tan Hospital, Beijing, China

The Steering Committee Members of NICE study are as follows: Yongjun Wang, Xingquan Zhao, Tao Feng, Bohua Lu, Rong Xue, Xiaoping Pan, Xiaoyuan Niu, Xu Zhang, Rubo Sui, En Xu.

The Data Safety and Monitoring Board includes: Yilong Wang，Yong Zhou, Hao Li, Haibo Wu, Gaifen Liu, Anxin Wang.

The Study Organization Executive Committee: Xingquan Zhao，Penglian Wang, Weixiong Shi, Yi Ju, , Wei Zhang

The Members of the Critical Events Committee are: Yansheng Li, Shanghai Renji Hospital, Shanghai, China; Anding Xu, The First Affiliated Hospital of Jinan University, Guangzhou, China; Peiyi Gao, Beijing Tian Tan Hospital, Beijing, China; Liping Liu, Beijing Tian Tan Hospital, Beijing, China.

Sub-center Principal Investigators by centers (In alphabetical order)

Beijing Puren Hospital, Bohua Lu; Beijing Tiantan Hospital, Capital Medical University, Yongjun Wang; Beijing Tongren Hospital, Capital Medical University, Xiaojun Zhang; Beijing Yanhua Phoenix Hospital, Zhengshe Bao; First Affiliated Hospital of Dalian Medical University, Dingbo Tao; First affiliated Hospital of Liaoning Medical University, Rubo Sui; First Hospital of Shanxi Medical University, Xiaoyuan Niu; Fu Xing Hospital Affiliated to Capital University of Medical Sciences, Guang Huang; Guangzhou First Municipal People's Hospital, Xiaoping Pan; Second Affiliated Hospital, Zhejiang University College of Medicine, Shuijiang Song; Second Artillery General Hospital of PLA, Lei Wang; The Affiliated Hospital of Medical College Qingdao University, Renliang Zhao; The First Affiliated Hospital of College of Medicine, Zhejiang University, Benyan Luo; The First Affiliated Hospital of Wenzhou Medical College, The first Provincial Wenzhou Hospital Zhejiang, Xu Zhang; The First Hospital of Fangshan District, Beijing, Jianhua Li; The First Hospital of Handan, Jie Lin; The First People's Hospital of Taizhou, Zhejiang Proveince, Zhimin Wang; The Friendship Hospital of Dalian City, Xiaohong Li; The Second Affiliated Hospital of Guangzhou Medical University, En Xu; The Second Affiliated Hospital of Harbin Medical University, Qingcheng Liang; Tianjin Medical University General Hosptial, Rong Xue; Wuhan Brain Hospital & Changhang General Hospital, Yuhua Chen; Xiangya Hospital,Central South University, Bo Xiao.
